# The MSH2 c.793-1G>A variant disrupts normal splicing and is associated with Lynch syndrome

**DOI:** 10.3389/fonc.2023.1131011

**Published:** 2023-07-19

**Authors:** Yiming Li, Lulu Yu, Jiajia Cui, Jiye Yin, Wei Wu

**Affiliations:** ^1^ Department of Geratic Surgery, Xiangya Hospital, Central South University, Changsha, Hunan, China; ^2^ Department of General Surgery, Xiangya Hospital, Central South University, Changsha, Hunan, China; ^3^ National Clinical Research Center for Geriatric Disorders, Xiangya Hospital, Central South University, Changsha, Hunan, China; ^4^ Department of Clinical Pharmacology, Xiangya Hospital, Central South University, Changsha, Hunan, China; ^5^ Institute of Clinical Pharmacology, Central South University, Hunan Key Laboratory of Pharmacogenetics, Changsha, Hunan, China; ^6^ Engineering Research Center of Applied Technology of Pharmacogenomics, Ministry of Education, Changsha, Hunan, China

**Keywords:** Lynch syndrome, mismatch repair gene, alternative splicing, MSH2 Lynch syndrome, MSH2, variant

## Abstract

**Instruction:**

Lynch syndrome (LS) is the most common inherited cancer predisposition disorder of colorectal cancer (CRC) which is associated with pathogenic variants in 4 mismatch repair (MMR) genes. Here, we reported a multi-generation Chinese family clinically diagnosed with LS.

**Methods:**

To identify the underlying pathogenic gene variants, 30 whole blood samples and 4 colorectal cancer tissue samples and their clinical data were obtained from this four-generation family. Microsatellite instability-high (MSI) testing, immunohistochemistry (IHC), and Whole-Exome Sequencing (WES) were performed to identify the MMR/MSI and the underlying gene variants. The minigene splicing assay and in vitro splicing assay were used to explore the function of this variant.

**Results:**

MSI-H and dMMR was revealed by the MSI testing and IHC, Whole-Exome Sequencing (WES) in 3 patients successfully identified a splicing variant (c.793-1G>A) in intron 4 of MSH2. Sanger sequencing validated the WES results, and all the “healthy” individuals carrying the variant have been identified in the family by PCR. Bioinformatics analysis and in vitro minigene assay showed that the pathogenic variant affected the splicing process of MSH2 gene to generate 2 kinds defective transcription products, and consequently reduced the expression of MSH2 protein. The mutation carriers were later recommended for colonoscopy and other important cancer diagnostic inspections every 1-2 years because they both have a higher risk of LS.

**Discussion:**

We found a pathogenic splicing variant (rs863225397, c.793-1G>A) of MSH2 gene, and furtherly confirmed that this mutation plays an important role in LS patients of this pedigree based on the vitro study. Our study indicates that one splicing mutation in the MSH2 gene (c.793-1G>A) causes LS and highlights the importance of LS gene testing.

## 1 Introduction

Globally, colorectal cancer (CRC) is one of the most prevalent cancers and has become a major public health problem, which ranks third as sorted by the new incidence and ranks second in terms of cancer-related death (1, 2). CRC is a multifactorial malignancy caused by both environmental and inherited factors playing varying roles in patients with CRC. Most CRCs arise as a consequence of somatic genomic events that disrupt key cellular processes in individual colonic epithelial cells (3), and, approximately, 15%–35% of CRCs are thought to be associated with hereditary factors (4, 5).

Lynch syndrome (LS), also known as hereditary non-polyposis CRC (HNPCC), is an autosomal dominant disease that markedly increases the lifetime risk of CRC, endometrial cancer (EC), and other cancers of the ovary, stomach, urothelial tract, small bowel, pancreas, biliary tract, and sebaceous neoplasms of the skin. LS is the most common cause of inherited CRCs and EC, and it accounts for 2.2%~5% of all patients with CRC and 3% of all patients with EC (6–9). Clinical criteria, tumor testing, and genetic testing are effective strategies for identifying patients with LS. As a commonly used testing method for LS, genetic testing confirms the diagnosis at the molecular level and provides a basis for the treatment plans, risk stratification, and surveillance plans for patients and career planning for families (10). Furthermore, extensive research studies that are concern about the genetic basis of LS have led to the novel concepts of tumor development and the feasibility of application to clinical diagnosis and treatment (11). Clinically, microsatellite instability (MSI) testing and mismatch repair (MMR) protein immunohistochemistry (IHC) tumor testing are the most commonly used examination assessments in LS-caused tumors (12). Research indicates that nearly all patients with LS have MSI/MMR-deficient tumors (13). MSI is a molecular hallmark of LS and represents germline mutations in one of the MMR genes (14). MMR genes play a pivotal role in the detection and rectifying of DNA sequence mismatches during DNA replication. Deficiency in MMR leads to MSI-H and hypermutability, resulting in a 100- to 1,000-fold increase in the mutation rate due to uncorrected base mismatches (15). MMR mutations occur in approximately 15% of CRCs and 9% of patients with EC. Carriers have a significantly higher lifetime risk of developing colorectal and ECs, as well as other tumors compared to the general population (16, 17). The majority of LS is caused by germline mutations in MMR genes, of which the four well-recognized MMR genes are mutL homolog 1 (MLH1), MSH2, MSH6, and postmeiotic segregation increased 2 (PMS2). Further identifying and characterizing the mutations might be crucial to enable personalized risk assessments of patients with LS (18).

Identification of a high-risk disease-causing constitutional mutation in a cancer patient guides the clinical management of the entire family, which has implications for counseling, cancer treatment options, pre-symptomatic surveillance, and consideration of risk-reducing surgery and/or medication regimes (19, 20). The sequencing of the MMR genes for mutations is the key procedure in diagnosing LS (7). MSH2 has the highest proportion of pathogenic variants, accounting for 40% of mutations found in LS families. The estimated cumulative risks of CRC by age 70 years reached 48%, and the risk of EC reached 21% (21). Although clinical evidence can contribute to evaluating the significance of these variants, usually, none of them can be used for clinically useful variant interpretation due to lack of laboratory evidence (19). Therefore, it is of great importance to export the pathogenicity and assess the risk of the variants for risk classification, treatment, prognostic monitoring strategy establishment, and genetic counseling. This will help identify individuals who would benefit from surveillance and prophylactic surgery to prevent cancer (22).

In this research, we identified a pathogenic MSH2 splicing site mutation in a four-generation LS family through whole-exome sequencing (WES). To determine the clinical significance of this variant, we analyzed the possible molecular pathogenesis and clinical phenotype of this family and provided appropriate individual prevention strategies for all mutation carriers.

## 2 Methods and materials

### 2.1 Patients and samples

The subjects included in this study were a four-generation family with LS in southern China. We collected 30 whole blood samples and four CRC tissue samples and their clinical data from Xiangya Hospital of Central South University (Changsha, Hunan, China) from April 2015 to August 2020. After a complete analysis of clinical information, we found that 30% (9 of 30) of the family members affected with the disease. This study was approved by the Ethics Committee of Xiangya School of Medicine, Central South University. Informed consent with the tenets of the Declaration of Helsinki was obtained from all participants or their guardians.

### 2.2 Whole-exome sequencing

Peripheral venous blood of III-14, and III-11, and III-15 was obtained before the surgical treatment of CRC. Genomic DNA was extracted from the whole blood sample. DNA fragments were sequenced on the NovaSeq 6000 high-throughput platform (Illumina, CA, USA). Single-nucleotide variant and insertion and deletion queries were performed as previously described (23, 24). The reference genome for WES was UCSC hg19, NCBI build 37.

### 2.3 DNA extraction and microsatellite instability analysis

Genomic DNA was isolated from blood samples of III-14 and III-15 and tested for MSI. A five-marker panel including two mononucleotide repeats (BAT25 and BAT26) and three dinucleotide repeats (D2S123, D5S346, and D17S250), which is recommended by the National Cancer Institute (NCI) Workshop on MSI for Cancer Detection and Familial Predisposition, was used as previously described (25). Oligonucleotide primers were fluorescently labeled, and PCR products were evaluated using the 3500DX Genetic Analyzer. Tumors were classified as highly unstable (MSI-H) if at least 40% of the markers showed instability (26).

### 2.4 Mismatch repair protein immunohistochemistry

MMR protein expression was tested by IHC using MLH1 polyclonal antibody clones (Proteintech Group, #11697-1-AP), MSH2 polyclonal antibody (Proteintech Group, #15520-1-AP), MSH6 polyclonal antibody (Proteintech Group, #18120-1-AP), and PMS2 polyclonal antibody (Affinity Group, #DF4351). The complete absence of protein expression of any of the four proteins tested (0+ in 100% of cells) was considered deficient MMR (dMMR). Formalin-fixed paraffin sections were stained for the abovementioned antibodies using the streptavidin-peroxidase system (Zhong-shan Goldenbridge Biotechnology, Beijing, China) as previously described (27).

### 2.5 Splicing prediction

Different complementary online software for splicing prediction were employed: Human Splicing Finder (HSF) (http://www.umd.be/HSF3/HSF.shtml), Mutation taster (http://www.mutationtaster.org/), NetGene2 (https://services.healthtech.dtu.dk), varSEAK (https://varseak.bio/index.php), and SpliceAI (https://spliceailookup.broadinstitute.org/).

### 2.6 Phylogenetic analysis

Phylogenetic analysis was performed using the Molecular Evolutionary Genetics Analysis (MEGA) software (www.megasoftware.net), and the evolutionary history was inferred by the maximum likelihood method, with 1,000 bootstrap values.

### 2.7 Construction of the minigenes and Sanger sequencing

A minigene splicing assay was performed to verify whether the mutation affected splicing products. Wild‐type (WT) or mutant-type (MT) plasmids encompassing exon 4, part of intron 4, and exon 5 of the MSH2 gene were constructed by amplification with genomic DNA from the affected patient II-9 using PrimeSTAR GXL DNA Polymerase (Takara, cat. no. R050Q). The amplified fragments were digested with Xho I (Takara, cat. no. 1094S) and EcoR I (Takara, cat. no. 1040S) and then subcloned into the multicloning sites of the pcDNA3.1 vector using Xho I and EcoR I restriction sites.

### 2.8 In vitro splicing assay

The WT and MT plasmids were transfected into Human Embryonic Kidney 293 cells (HEK293) and Hela cells using the PolyJet Transfection kit (SignaGen Laboratories, MD, USA). After 48 h, RNA was extracted and reverse-transcribed into complementary DNA (cDNA). Minigene constructs were validated (with PCR products from exon 4 to exon 5) by Sanger sequencing analysis with the universal primer (5′‐TATGTTTCAGGTTCAGGG‐3′; Sangon Biotech).

## 3 Results

### 3.1 A four-generation Chinese family with Lynch syndrome

**Figure 1:**
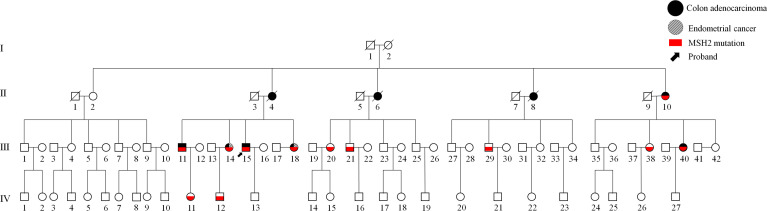
A four-generation Chinese family with Lynch syndrome (LS). Squares represent men and circles represent women. A square or circle with a diagonal line means dead, and vice versa means alive. Filled black symbols indicate members affected with colon cancer, whereas filled slant lines indicate members affected with endometrial carcinoma and empty symbols are unaffected family members, and the red symbols indicate the c.793-1G>A mutation in MSH2. The black arrow indicates the proband (III-15).

We identified a four-generation Chinese pedigree with 81 members, of whom nine (II-4, II-6, II-8, II-10, III-11, III-14, III-15, III-18, and III-40) were affected with colon cancer, and two (III-14 and III-18) were affected with EC followed by colon cancer (**Figure 1**). The proband, a 43-year-old man (III-15), was diagnosed with left colonic carcinoma *via* colonoscopy and biopsy. No other abnormalities or obstructions were observed. He subsequently underwent formal resection of left hemicolon cancer and anastomosis, with postoperative pathologic examination confirmed a poorly differentiated, T4N2 adenocarcinoma. His younger sister, a 39-year-old woman (III-18), was affected with endometrial carcinoma 6 years ago and diagnosed with right side colon carcinoma in a routine colonoscopy without specific symptoms. She subsequently underwent subtotal colectomy with ileo-rectal anastomosis, hysterectomy, and bilateral salpingo-oophorectomy. The tumor was then identified as moderately differentiated adenocarcinoma staging at T4N0, with no abnormalities detected in the uterus or bilateral salpingo-oophorectomy.

The elder sister (III-14) of the aforementioned two patients was diagnosed with left hemicolon cancer at the age of 37 and subsequently underwent a limited colectomy. Ten years later, she received a transabdominal hysterectomy and bilateral salpingo-oophorectomy due to endometrial carcinoma. Shortly thereafter, a sessile polyp was discovered in her transverse colon during a colonoscopy. Upon our recommendation, she underwent subtotal colectomy with ileorectal anastomosis. The postoperative pathological examination reported an intestinal mucous adenocarcinoma, with invasion to the submucosa (T1N0). As evidenced by their family history, in addition to the abovementioned three patients, colon cancer has also affected their eldest brother, mother, and three out of the four maternal aunts. These patients have been definitively diagnosed with LS according to the Amsterdam II/III criteria. The detailed information of affected members or mutation carriers in this pedigree is shown in **Table 1**.

**Table 1:** Clinical characteristics of all the affected and mutation carriers of the LS family.

Family ID	Sex	Age	Mutation	Age at Diagnosis	Colonic diagnosis (Diagnosed Age)	Extra-Colonic diagnosis (Diagnosed Age)
II-4	F	48(Deceased)	Unknown		colonic carcinoma(uncertain)	
II-6	F	53(Deceased)	Unknown		colonic carcinoma(uncertain)	
II-8	F	57(Deceased)	Unknown		colonic carcinoma(uncertain)	
II-10	F	73	MT	Lynch Syndrome (49)	Adenocarcinoma (49)	
III-11	M	56	MT	Lynch Syndrome (46)	Adenocarcinoma (46)	
III-14	F	54	MT	Lynch Syndrome (37)	Adenocarcinoma (37)	Endometrial carcinoma (47)
III-15	M	50	MT	Lynch Syndrome (43)	Adenocarcinoma (43)	
III-18	F	46	MT	Lynch Syndrome (39)	Adenocarcinoma (31)	Endometrial carcinoma (31)
III-20	F	52	MT	Not yet penetrant		
III-21	M	50	MT	Not yet penetrant		
III-29	M	48	MT	Not yet penetrant		
III-38	F	43	MT	Not yet penetrant		
III-40	F	46	MT	Lynch Syndrome (44)	Adenocarcinoma (44)	
IV-11	F	31	MT	Not yet penetrant		
IV-12	M	30	MT	Not yet penetrant		

### 3.2 MSI-H was found in the pedigree

**Figure 2:**
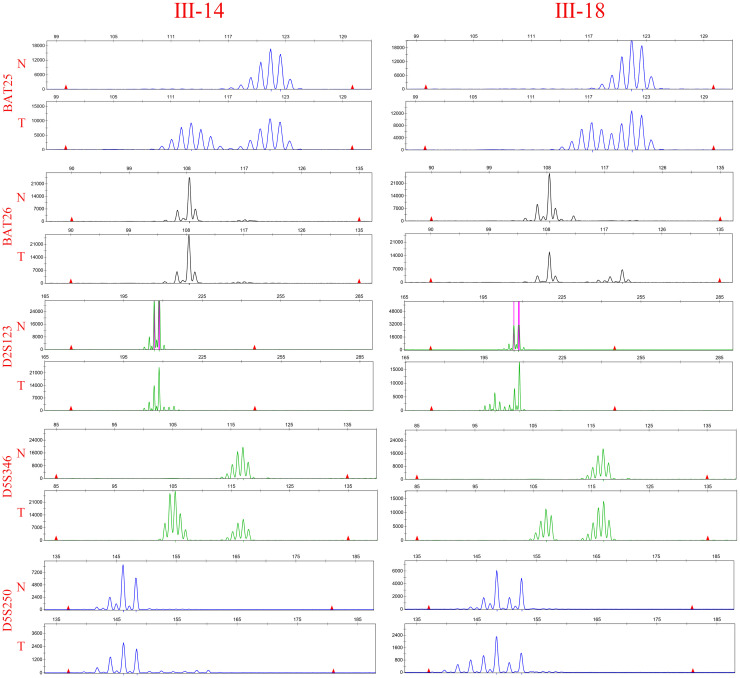
MSI-H was found in the pedigree. Microsatellite analysis of the five reference markers BAT25, BAT26, D5S346, D2S123, and D17S250 in III-15 and III-18. Tumor DNA (lane below) reveals new bands and a band shift compared with the corresponding genomic DNA (lane above).

A number of studies have indicated that screening for LS is imperative in patients with CRC, regardless of their age or pathological stage (10, 28, 29). The identification of MSI, which primarily relies on PCR analysis, serves as a crucial biomarker in the management of CRC. Therefore, tumor and normal tissues from patients III-15 and III-18 were obtained after surgery to assess MSI status. For the five detection markers [two mononucleotides loci (BAT25 and BAT-26) and three dinucleotide loci (D2S123, D17S250, and D5S346)] recommended by of the NCI of the United States and National Comprehensive Cancer Network (NCCN) guidelines, the detection results are shown in **Figure 2**. The findings indicate that patients III-15 and III-18 are in the MSI-H state, which is a predictive marker for resistance to 5-fluorouracil (5-Fu); however, immune checkpoint blockades have demonstrated durable response and sustained survival benefits (30).

### 3.3 The deficiency of MMR was found in the pedigree

**Figure 3:**
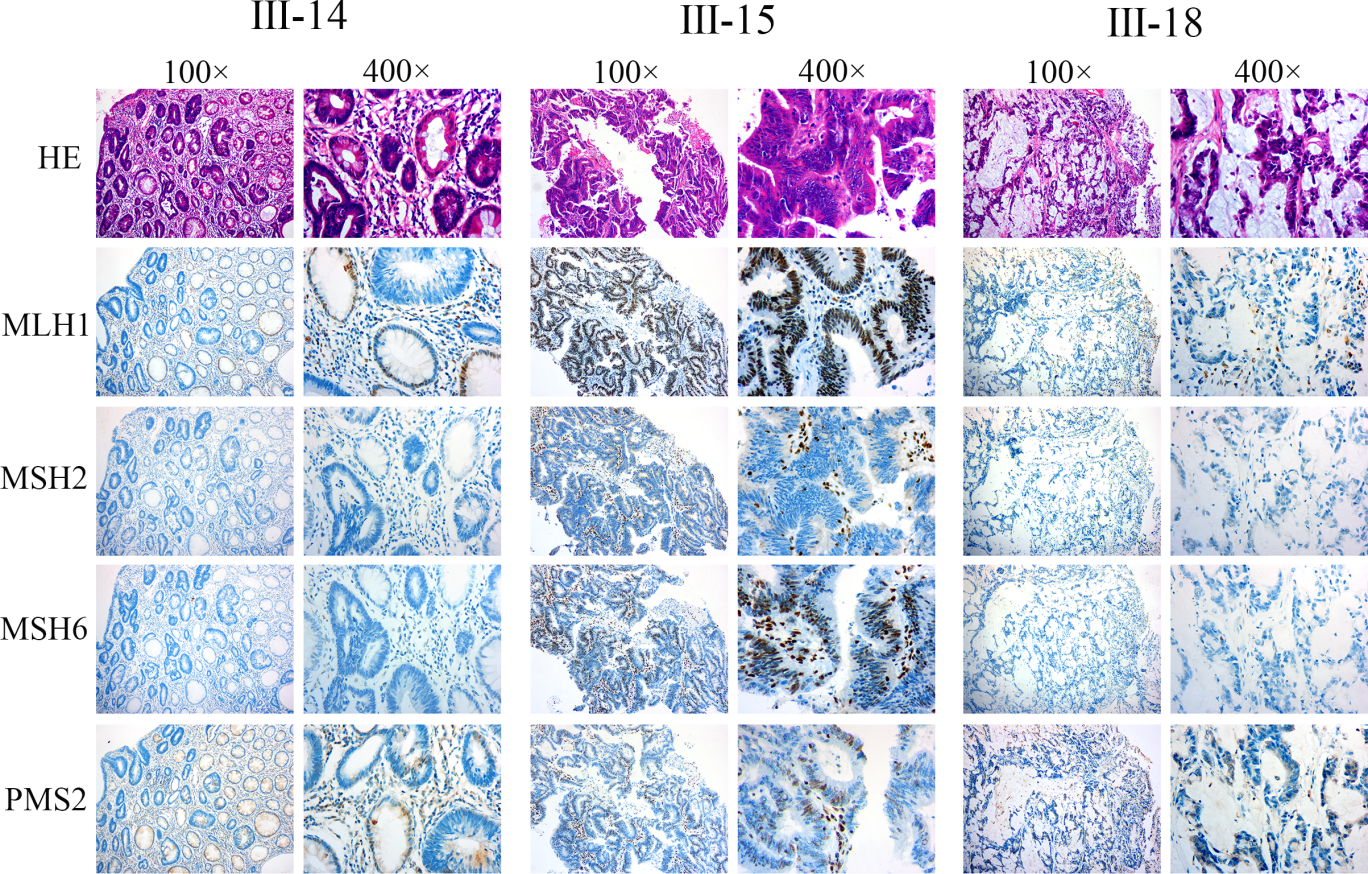
The deficiency of MMR was found in the pedigree. H&E and IHC staining was conducted on the serial paraffin embedding tumor tissue sections of III-14, III-15, and III-18. The results indicate III-14: intestinal mucous adenocarcinoma, MLH1+, MSH2−, MSH6−, and PMS+; III-15: poorly differentiated adenocarcinoma, MLH1+, MSH2−, MSH6+, and PMS+; III-18: moderately differentiated adenocarcinoma, MLH1+, MSH2−, MSH6−, and PMS+.

As described above, we determined MSI status by amplification of specific microsatelilte repeats. To explore the underlying cause of MSI-H states and confirm the above result of MSI detection, we also detected the expression of MMR-related proteins (MLH1, MSH2, MSH6, and PMS2) to further confirm the presence of MMR defects. Hematoxylin-eosin (H&E) staining and IHC were performed on the serial paraffin-embedded tumor tissue sections of III-14, III-15, and III-18. The results revealed defective MSH2 and MSH6 in the tumor nuclei of III-14 and III-18, whereas only MSH2 was defective in tumor nuclei of III-15 (**Figure 3**), thus confirming the dMMR status in III-14, III-15, and III-18. Previous research has described that MSH2 can dimerize with MSH6, and the MSH2 mutation often leads to in deficiency in both MSH2 and MSH6 protein expression, whereas MSH6 gene mutation typically result in loss of MSH6 protein expression (31, 32); based on our IHC results, it is suggested that LS of this pedigree might be caused by MSH2 mutation.

### 3.4 A pathogenic splicing variant in MSH2 was found in the pedigree

**Figure 4:**
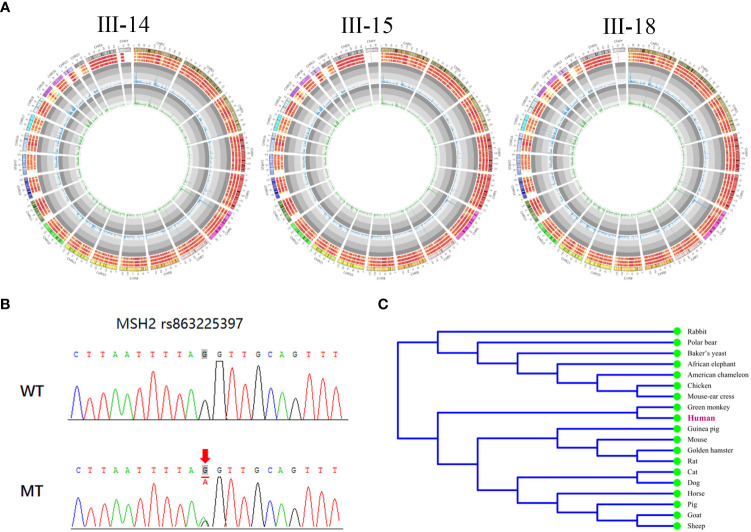
A pathogenic splicing variant in MSH2 was found in the pedigree. **(A)** The landscape of all types of variations in the three probands. The columns in the first circle indicated the number of SNVs per Mb. The second circle indicated the number of variations (SNV + indel) per Mb. The three circles outside indicated the number of indels per Mb, the number of SNVs per Mb, and the number of variation per Mb. **(B)** Sanger sequencing of the MSH2 gene showing a pathogenic splicing mutation c.793-1G>A in the affected members in the pedigree. **(C)** The conservation of the mutation in MSH2 across species.

To investigate the genetic factors underlying LS in this family, WES was performed on individuals III-14, III-15, and III-18. All variations are shown in **Figure 4A**. We focused our analysis on functionally significant mutations located within exonic regions and splicing sites.

A high frequency of variants was found in genes at high risk of LS. In III-14, the total number of germline SNVs and InDels was 42,933 and 3,219, respectively. In III-15, they were 42,731 and 3,161, respectively; in III-18, the numbers were 42,925 and 3,193, respectively. We also found mutations located in the coding and splice regions of all these genes [MLH1, MSH2, MSH6, PMS2, epithelial cell adhesion molecule (EPCAM), mutY homologue (MUTYH), Phosphatase and tensin homolog (PTEN)] that may be associated with the pathogenesis of LS depending on their mutation type. Among these mutations, the genetic variant occurring in all three patients is what we looking for. One same splicing mutation (c.793-1G>A) was found in the three patients, which might be the heterozygous pathogenic variant and have not previously described in the MSH2 gene [NCBI reference sequence NM_000251.2]. The basic information of this mutation is shown in **Table 2**, and Sanger sequencing confirmed this mutation in three family members (**Figure 4B**).

**Table 2:** Basic information of splicing mutation c.793-1G>A in MSH2.

SNP	Position (GRCh38.p12)	Gene	Frequency in gnomAD	Mutation mode	Mutation type	cDNA alteration	Protein alteration
rs863225397	Chr2:47414268	MSH2	—	Het	Splice Acceptor variant	c.793-1G>A	——

Furthermore, we identified that this mutation is located at the splicing donor site and disrupts the normal splicing of the pre-mRNA, resulting in an aberrant MSH2 protein. In addition, through utilization of MEGA software, we observed high conservation of this mutation across multiple species (**Figure 4C**). These results indicate that the mutation may play an essential role in the function of the MSH2 protein.

### 3.5 Functional prediction of the intronic variant C.793-1G>A

The intronic variant C.793-1G>A is located in a highly conserved 3′ splicing acceptor site. To assess the impact of the c.793-1G>A mutation on splicing process in silico, we analyzed WT and MT 3′ splice site (3SS) located in the intron 4 using the HSF, NetGene2, varSEAK, and SpliceAI prediction websites. As shown in **Table 3**, whereas all four programs recognized the WT 3SS as an acceptor site, none identified MT 3SS as such.

**Table 3:** Bioinformatic prediction of the effect of the c.793-1G>A mutation on splicing process.

Intron	Nucleotide change	5’ Splice site	Bioinformatic splicing prediction						
			HSF		SpliceAI		VarSEAK		NetGene2
Intron4	c.793-1G>A	ttaattttagGTTGCA →ttaattttaaGTTGC A	WT site broken	10.35-1.6 (-84.54%)	WT site Loss	1	WT site broken	-39.51%	WT site broken
			NEW site	-2.15-5.8 (+369.77%)	NEW site gain	0.92	NEW site	11.28%	

The HSF algorithm showed a ΔCV of −32.13% (CV WT = 86.74 vs. CV mutant = 58.87). Furthermore, MaxEnt, the MaxEnt algorithm for HSF, also demonstrated a ΔCV of −84.54%. On the basis of the SpliceAI online tool, we observed that the variant results in a loss of confidence score for the original splicing site and generates a new site with its confidence score. Both HSF and SpliceAI algorithms indicate that the delta coefficients of variation (ΔCV) values suggest disruption of the acceptor site, which probably activates an intronic cryptic acceptor site, suggesting that the mutation affects splicing.

**Figure 5:**
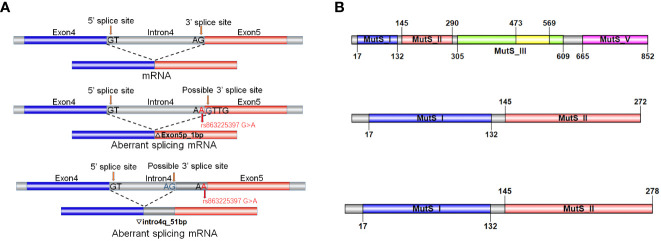
Functional prediction of the intronic variant C.793-1G>A. **(A)** Schematic representation of splicing models in bioinformatic analysis. The normal MSH2 gene transcribing and splicing process to generate complete mRNA. The variant causes the loss of the original splicing site and may generate a new splicing site and then construct two different types of aberrant splicing mRNA product. **(B)** Schematic representation of translation by the normal and aberrant splicing products. The two aberrant splicing mRNA products resulting in truncated proteins of 272aa and 278aa in length, respectively.

The online tool varSEAK not only predicts the generation of a new splicing site but also further predicts two scenarios: 1-bp deletion to the left of exon 5 or 51-bp retention to the right of intron 4 (**Figure 5A**). In contrast, NetGene2 only predicts the disappearance of the original acceptor site. Functional prediction of this variant was conducted through bioinformatic tool to predict splicing signals. The results indicate that this variant leads to loss of the original splicing site and may generate a new splicing site and then construct two different types of aberrant splicing mRNA.

We conducted further protein-level analysis of the two aberrant splicing products mentioned above. After a 1-bp deletion on the left side of exon 5, c.793del p.Val265Leufs*9 generates an early termination codon PTC in exon 5 that may undergo nonsense-mediated mRNA horizontal degradation, leading to a truncated protein of 272 amino acid (aa) in length. After intron 4 right lagging 51 bp, c.792_793ins51bp p.Val265Asnfs*15 in intron 4 produces the early termination codon PTC, which may also produce a further truncated protein of 278aa in length (**Figure 5B**). We hypothesized that this variant may impact the splicing of MSH2, leading to aberrant protein structure and function, thereby contributing to the development of LS in this family.

### 3.6 Function study verified the aberrant splicing products caused by C.793-1G>A mutation

**Figure 6:**
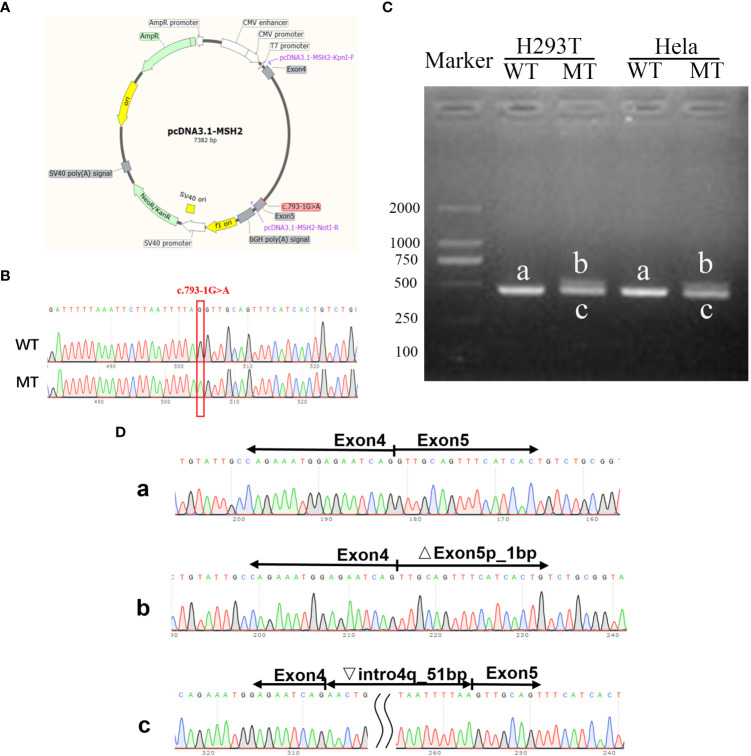
Function study verified the aberrant splicing products caused by C.793-1G>A mutation. **(A)** Schematic illustration of the pcDNA3.1 MSH2 c.793-1G>A minigene. **(B)** Sanger sequencing confirmed the successful construction of wt and mt plasmids. **(C)** Gel electrophoresis of the RT-PCR product of minigene transcripts in HEK293T and Hela cells. **(D)** Sanger sequencing analysis of alternatively splicing products. The letter a, b, c means the RT-PCR product of the WT or MT plasmids.

To analyze the effect of the intron donor site mutation on the MSH2 mRNA splicing, an in vitro transcription assay was conducted. The splicing pattern of WT and MT plasmids was illustrated (**Figure 6A**) and confirmed by Sanger sequencing (**Figure 6B**). The WT minigene is 2,005 bp long and encompasses DNA regions comprising exon 4 (147 bp), part of intron 4 (1,708 bp), and exon 5 (150 bp). A full-length 457-bp Real Time (RT)--PCR product (partial plasmid sequence 160 bp and target gene 297 bp, named band a) was expressed and detected in HEK293 cells, which includes: exon 4 and exon 5, as expected (**Figure 6C**).

The sequencing results revealed that WT band a was generated through normal splicing, whereas band b had 1-bp deletion on the left side of exon 5, and band c retained 51 bp on the right side of intron 4 (**Figure 6D**). The same result was observed in Hela cells, which is consistent with the results predicted by the varSEAK tool.

Consistent with previous bioinformatic prediction, the minigene construct containing the C.793-1G>A mutation produced two predominant RT-PCR products. In one case, band b exhibited a size similar to the WT band a. This was owing to the mutation producing a new acceptor site (consisted of the A mutated from G at the end of intron 4, with the G at start of exon 5 coincidently) on the left side of exon 5, resulting in a 1-bp deletion on the left side of exon 5. In the other situation, a new acceptor site (a new AG motifs was recognized at 51 bp from end intron 4) was identified on the right side of intron 4, resulting in a 51-bp extension on the right side of intron 4 and yielding a significantly larger band c compared to the WT band a. This finding corroborated our bioinformatic predictions.

## 4 Discussion

LS is a well-established inherited condition caused by defective DNA MMR, and the mean age of CRC diagnosis in patients with LS is 44~61 years (7, 10, 33). Recent research has shown that patients with LS are typically diagnosed with CRC at an average age of 50.7 (34). The MMR gene group comprises crucial genes that maintain genome stability in cells. Defects in these genes lead to the failure of timely and effective correction of oncogene-related mutations, thereby promoting tumor susceptibility. Among the MMR genes, MSH2 is the most commonly occurring variant gene, and splicing represents the least variable type among MSH2 mutations (35). In this research, we have identified a pathogenic germline splicing site mutation.

As the NCCN guidelines recommended, LS evaluation should be considered for patients with a PREMM1,2,6 score of ≥5%. Actually, the proband and the other two patients all scored ≥50% in PREMM1,2,6; therefore, it is imperative to screen for LS. Subsequently, the PCR-based MSI detection and IHC-based MMR protein expression analysis was performed. These results were consistent with the WES, all indicating that the disease-causing mutation locates at MSH2.

In this four-generation family, we identified a splicing site mutation C.793-1G>A in MSH2 by WES. Previously, this mutation was just listed in a Swedish study among 369 mutations without any further description (36). Interestingly, we suppose that this mutation might happen in multi races, and it is worthy of more attention. In our research, we found the dominant inherited pathogenic mutation in southern China; more than that, certain bioinformatic analysis reveals that this mutation site was relatively conserved among species. Previous research has described that certain MMR protein expression deficiencies indicate a pathogenic mutation of the corresponding MMR genes. In this research, IHC confirmed that MSH2 and MSH6 were defective in III-14 and III-18, and MSH2 was defective in III-15, both indicating pathogenic MSH2 mutation. The MSI testing also proved the MSI-H of III-15 and III-18, which was caused by the functional deficiency of MSH2.

The majority of MMR gene variants detected in patients with LS are truncating mutations, which are generally classified as pathogenic (21). Conserved sites at exon/intron junctions or in the introns play a critical role to ensuring proper splicing. Mutations affecting these conserved splicing sites may result in abnormal RNA splicing products, leading to dysfunctional protein products and ultimately causing disease (37). Previously, a germline variant c.2635-3delC within the splice acceptor site of exon 16 in the MSH2 gene, which affects normal splicing and might be a cause of LS (38). Similarly, multiple variants of splicing acceptor located in dinucleotides at each end of the intron have been found in the MSH2 gene (39) but have not yet been identified in multiple family members in a relatively large pedigree and also lack identification in cancer cells through plasmid transfect. Through this research, we have identified a mutation located at the acceptor splicing site of intron 4 and close to exon 5 within the MSH2 gene. This mutation has the potential to significantly impact normal splicing of MSH2. To investigate this further, we conducted a Minigene experiment by constructing a plasmid using pcDNA3.1 as the carrier, transfected it into HEK293 cells and Hela cells, respectively. We then extracted RNA samples to determine the effect on gene expression. Our results showed that mutation c.793-1G>A will affect the normal splicing of mRNA and may produce two aberrant RNA splicing: 1-bp deletion on the left side of exon 5 and 51-bp retention on the right side of intron 4, which may produce two kinds of truncated protein. As indicated by the MMR gene variant classification criteria (https://www.insight-group.org/criteria/), the mutation c.793-1G>A in MSH2 definitely results in a splicing aberration, and disrupting MSH2 protein expression should be classified to class 5 (pathogenic). In the future, we will further investigate the impact of this mutation on MSH2 protein domain and function, as well as elucidate the precise mechanism underlying LS.

In this research, we have noticed that the C.793-1G>A mutation is a pathogenic variant and might cause relatively early-onset cancer and is susceptible to EC in female mutation carriers. Given these characteristics, we recommended genetic testing for most family members and pay close attention to the mutation carriers. For the carriers, we prefer earlier and more regularly recommended colonoscopy every 1 year beginning at age 20 for IV-11 and IV-12. The female carriers were educated regarding the symptoms of endometrial and ovarian cancer, and the endometrial biopsy and EC screening were recommended every 1 year beginning at 30 (III-20, III-38, III-40, and IV-11). Risk-reducing surgery is also applicable for patients with LS or mutation carriers at high-risk ages (6). Individuals with LS who develop CRC are recommended to undergo a total abdominal colectomy with an ileorectal anastomosis due to the high risk for second primary CRCs as well as prophylactic hysterectomy and bilateral salpingo-oophorectomy in female carriers due to the risk of endometrial and ovarian cancer (33, 40).

In summary, these findings provide some clinical and functional evidence that the C.793-1G>A variant of MSH2 gene can contribute to LS and can be further studied as a potential target for the diagnosis and treatment of colon cancer.

## Data availability statement

The data presented in the study are deposited in the SRA repository, the related BioProject accession number PRJNA993972, and the link is at http://www.ncbi.nlm.nih.gov/bioproject/993972.

## Ethics statement

This study is conducted in accordance with the Declaration of Helsinki, and the involving human participants were reviewed and approved by Ethics Committee of Xiangya Hospital, Central South University. Written informed consent for participation was not required for this study in accordance with the national legislation and the institutional requirements.

## Author contributions

YL and WW conceived the study and wrote the manuscript. YL and LY conducted the experiments and contributed to the data analysis. YL, LY, JC, JY, and WW collected clinical samples and corresponding clinical data. YL, LY, and WW revised the manuscript. All authors contributed to the article and approved the submitted version.
